# Esmolol during cardiopulmonary resuscitation reduces neurological injury in a porcine model of cardiac arrest

**DOI:** 10.1038/s41598-021-90202-w

**Published:** 2021-05-20

**Authors:** Laura Ruggeri, Francesca Nespoli, Giuseppe Ristagno, Francesca Fumagalli, Antonio Boccardo, Davide Olivari, Roberta Affatato, Deborah Novelli, Daria De Giorgio, Pierpaolo Romanelli, Lucia Minoli, Alberto Cucino, Giovanni Babini, Lidia Staszewsky, Davide Zani, Davide Pravettoni, Angelo Belloli, Eugenio Scanziani, Roberto Latini, Aurora Magliocca

**Affiliations:** 1 Istituto Di Ricerche Farmacologiche Mario Negri IRCCS, Milan, Italy; 2 Department of Pathophysiology and Transplantation, University of Milan, Milan, Italy; 3 Department of Anesthesiology, Intensive Care and Emergency, Fondazione IRCCS Ca’ Granda Ospedale Maggiore Policlinico, Via Francesco Sforza 35, 20122 Milan, Italy; 4 Dipartimento Di Medicina Veterinaria, University of Milan, Lodi, Italy; 5 Mouse and Animal Pathology Lab (MAPLab), Fondazione UniMi, Milan, Italy

**Keywords:** Ventricular fibrillation, Preclinical research

## Abstract

Primary vasopressor efficacy of epinephrine during cardiopulmonary resuscitation (CPR) is due to its α-adrenergic effects. However, epinephrine plays β1-adrenergic actions, which increasing myocardial oxygen consumption may lead to refractory ventricular fibrillation (VF) and poor outcome. Effects of a single dose of esmolol in addition to epinephrine during CPR were investigated in a porcine model of VF with an underlying acute myocardial infarction. VF was ischemically induced in 16 pigs and left untreated for 12 min. During CPR, animals were randomized to receive epinephrine (30 µg/kg) with either esmolol (0.5 mg/kg) or saline (control). Pigs were then observed up to 96 h. Coronary perfusion pressure increased during CPR in the esmolol group compared to control (47 ± 21 vs. 24 ± 10 mmHg at min 5, *p* < 0.05). In both groups, 7 animals were successfully resuscitated and 4 survived up to 96 h. No significant differences were observed between groups in the total number of defibrillations delivered prior to final resuscitation. Brain histology demonstrated reductions in cortical neuronal degeneration/necrosis (score 0.3 ± 0.5 vs. 1.3 ± 0.5, *p* < 0.05) and hippocampal microglial activation (6 ± 3 vs. 22 ± 4%, *p* < 0.01) in the esmolol group compared to control. Lower circulating levels of neuron specific enolase were measured in esmolol animals compared to controls (2[1–3] vs. 21[16–52] ng/mL, *p* < 0.01). In this preclinical model, β1-blockade during CPR did not facilitate VF termination but provided neuroprotection.

## Introduction

The mainstays of cardiac arrest (CA) treatment are represented by early initiation of high-quality cardiopulmonary resuscitation (CPR) and prompt defibrillation, when appropriate^1^. Initial pharmacological interventions during CPR are directed to raise coronary perfusion pressure (CPP) by increasing peripheral vascular resistance, with the intent to improve the chances for return of spontaneous circulation (ROSC)^1–3^. Thus, epinephrine has been the recommended adrenergic agent during CPR for more than 50 years, although its efficacy in improving CA outcome has not been unanimously demonstrated^1,4–7^. A recent large randomized controlled trial confirmed the benefits of epinephrine on ROSC and survival to hospital discharge, while no effects on survival with favorable neurological outcome were reported^7^.

The primary vasopressor efficacy of epinephrine is due to its α-adrenergic effects on vascular smooth muscle, which cause peripheral vasoconstriction during CPR. However, epinephrine also has potent β1-adrenergic agonist actions, which increase myocardial oxygen utilization, failing to improve the balance between oxygen supply and demand^8–10^. This detrimental effect may worsen post-CA myocardial dysfunction and increase the sensitivity of the myocardium to arrhythmias, leading to refractory ventricular fibrillation (VF)^8,11^. Indeed, a recent metanalysis anticipated potential beneficial effects of esmolol, an ultrashort-acting β1-blocker, on short and long-term outcome in CA patients with refractory VF^12^.

In animal studies on CA, β1-adrenergic blockade with esmolol administered with epinephrine during CPR has been shown to reduce myocardial dysfunction and to increase the rate of ROSC with fewer defibrillations and survival, when compared to epinephrine alone^13–16^. Nevertheless, all these investigations were conducted in models of electrically induced VF in normally perfused hearts until CA. Recently, administration of esmolol, during veno-arterial extracorporeal membrane oxygenation resuscitation failed to demonstrate a clear cardioprotective effect or improvement in outcome in an ischemically-induced CA^17^.

Thus, the beneficial effects of esmolol administration during CPR in preventing the adverse β1-adrenergic effects of the epinephrine should also be confirmed in the case of a CA due to ischemic origin, i.e. due to acute myocardial infarction (AMI). The aim of this study was to investigate the effects of a single dose of esmolol, compared to placebo, in addition to epinephrine during CPR in a preclinical porcine model of CA with an underlying AMI^18–20^. A decrease in the total number of defibrillations needed to terminate VF with esmolol compared with placebo was hypothesized.

## Methods

**Figure 1:**
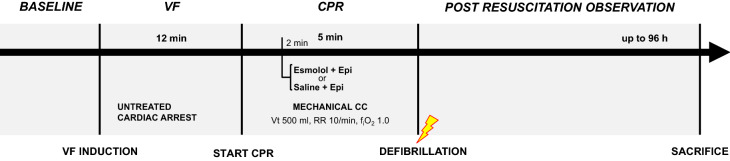
Study design. VF, ventricular fibrillation; CPR, cardiopulmonary resuscitation; CC, chest compression; RR, respiratory rate; Epi, epinephrine.

This was a randomized, placebo-controlled experimental study investigating the effects of esmolol (Brevibloc, Baxter, IL, USA) compared to placebo (saline, NaCl 0.9%) in addition to epinephrine during CPR in a porcine ischemic CA model. The study design is detailed in Fig. 1.

All procedures involving animals and their care were in conformity with national and international laws and policies. Approval of the study was obtained by the University of Milan Institutional review board committee and Governmental Institution (Italian Ministry of Health: approval no. 72/2014-PR). The study is reported in accordance with the ARRIVE (Animal Research: Reporting of In Vivo Experiments) guidelines.

### Animal preparation

Sixteen healthy male domestic pigs (39 ± 0.5 kg) were fasted the night before the experiment except for free access to water. Anesthesia was induced by intramuscular injection of ketamine (20 mg/kg) followed by intravenous administration of propofol (2 mg/kg) and sufentanyl (0.3 μg/kg) through an ear vein access. Anesthesia was then maintained by continuous intravenous infusion of propofol (4–8 mg/kg/h) and sufentanyl (0.3 μg/kg/h). A cuffed tracheal tube was placed, and animals were mechanically ventilated with a tidal volume of 15 mL/kg and FiO_2_ of 0.21. Respiratory frequency was adjusted to maintain the end-tidal partial pressure of carbon dioxide (EtCO_2_) between 35 and 40 mmHg, monitored with an infrared capnometer^18–20^. To measure aortic pressure, a fluid-filled 7F catheter was advanced from the right femoral artery into the thoracic aorta. For measurements of right atrial pressure (RAP), core temperature, and left ventricular (LV) cardiac output (CO), a 7F pentalumen thermodilution catheter was advanced from the right femoral vein into the pulmonary artery. Conventional pressure transducers were used (MedexTransStar, Monsey, NY). Myocardial infarction was induced in a closed-chest preparation by intraluminal occlusion of the left anterior descending (LAD) coronary artery^18–20^. Briefly, a 6F balloon-tipped catheter was inserted from the right common carotid artery and advanced into the aorta and then into the LAD, beyond the first diagonal branch, with the aid of image intensification and confirmed by injection of radiographic contrast media. For inducing VF, a 5F pacing catheter was advanced from the right jugular vein into the right ventricle. The position of all catheters was confirmed by characteristic pressure morphology and/or fluoroscopy. A frontal plane electrocardiogram was recorded.

### Experimental procedure

Fifteen min prior to inducing CA, animals were randomized by the sealed envelope method to receive at min 2 of CPR one of the following intravenous treatments in addition to epinephrine (30 µg/kg): (1) esmolol 0.5 mg/kg; or (2) equivalent volume of normal saline (control).

After baseline measurements, the LAD catheter balloon was then inflated with 0.7 mL of air to occlude the flow, as previously described^18–20^. If VF did not occur spontaneously after 10 min, CA was induced with 1 to 2 mA AC current delivered to the right ventricle endocardium. Ventilation was discontinued after the onset of VF. After 12 min of untreated VF, CPR, including chest compressions with the LUCAS 2 (PhysioControl Inc, Lund, Sweden) and ventilation with oxygen (tidal volume of 500 mL, 10 breaths/min), was initiated. At min 2 of CPR, animals received central venous bolus administration of either esmolol or saline, immediately followed by epinephrine. After 5 min of CPR, defibrillation was attempted with a single biphasic 150-J shock, using an MRx defibrillator (Philips Medical Systems, Andover, MA). If ROSC was not achieved, CPR was resumed and continued for 1 min before a subsequent defibrillation. Additional doses of adrenaline were administered at min 7 and 12 of CPR. The resuscitation maneuvers were continued until successful ROSC or for a maximum of 15 min. ROSC was defined as the restoration of an organized cardiac rhythm with a mean arterial pressure (MAP) of more than 60 mmHg. After that, if VF reoccurred, it was treated by immediate defibrillation. Immediately after resuscitation, the LAD catheter correct placement was reconfirmed by fluoroscopy^20^. After successful resuscitation, anesthesia was maintained, and animals were invasively monitored for 4 h. Forty-five minutes after resuscitation, the LAD catheter was withdrawn. Temperature of the animals was maintained at 38 °C ± 0.5 °C during the whole experiment. After 4 h, catheters were removed, wounds were repaired, and the animals were extubated and returned to their cages. Analgesia with butorphanol (0.1 mg/kg) was administered by intramuscular injection. At the end of the 96 h post-resuscitation observational period, animals were sacrificed painlessly with an intravenous injection of 150 mg/kg sodium thiopental, and heart and brain were harvested for histology.

### Measurements

Hemodynamics, EtCO_2_, and electrocardiogram were recorded continuously on a personal computer-based acquisition system (WinDaq DATAQ Instruments Inc, Akron, OH). CPP was computed from the differences in time-coincident diastolic aortic pressure and right atrial pressure. CO was measured by thermodilution technique (COM-2; Baxter International Inc, Deerfield, IL). Arterial blood gases were assessed with an i-STAT System (Abbott Laboratories, Princeton, NJ). Plasma high-sensitivity cardiac troponin T (hs-cTnT) and serum neuronal specific enolase (NSE) were measured with electrochemiluminescence assays (Roche Diagnostics Italia, Monza, Italy).

As previously described^18–20^, neurologic recovery was assessed with the neurologic alertness score (NAS), ranging from 100 (normal) to 0 (brain death), and with the swine neurologic deficit score (NDS), ranging from 0 (normal) to 400 (brain death). Finally, the functional recovery was evaluated before the sacrifice according to overall performance categories (OPCs) as follows: 1 = normal, 2 = slight disability, 3 = severe disability, 4 = coma, and 5 = brain death or death. Outcome was defined as poor when OPC was ≥ 3. Scores were assessed by veterinarians blinded to treatment.

At sacrifice, the brains were carefully removed from the skulls and fixed in 4% buffered formalin. Standardized 5-mm coronal slices were taken. The hippocampal CA1 sector and the cortex were chosen as regions of interest and were paraffin embedded. Five-micrometer-thick sections were then obtained and stained with hematoxylin–eosin. The proportion of neuronal loss and degeneration/necrosis (shrunken neurons with deeply acidophilic cytoplasm and pyknotic nucleus) was quantified as absent (0), rare (1), few (2), and numerous (3). Immunohistochemistry with antibody against microglia-specific ionized calcium-binding adaptor molecule 1 (Iba1) was used to detect reactive microglia activation. The evaluation was performed in the pyramidal CA1 layer of the hippocampus. Three fields, corresponding to an area of 0.3 mm^2^ each, were chosen among the most positively stained (hot spots) and photographed. Digital microphotographs of Iba1-immunostained sections were submitted to a semiautomated image analysis (ImageJ analysis program http://rsb.info.nih.gov/ij/). The area of reactive microglia was expressed as a percentage of Iba1-positive stain^18–20^. An experienced pathologist, blinded to treatment, performed the assessments.

Myocardial infarct was assessed by tetrazolium chloride (TTC) staining. The LV was sliced into 5-mm-thick transverse sections, which were incubated (20 min) in a TTC solution and then transferred to 4% formalin overnight before image analysis. Infarct size was reported as the percentage of TTC-negative area relative to LV area^18–20^.

### Statistical analysis

One sample Kolmogorov–Smirnov Z test was used to confirm the normal distribution of the data. Categorical variables were presented as proportion, while continuous variables as mean ± SEM or median [Q1–Q3], as appropriate. Differences in clinical characteristics according to experimental group (control vs. esmolol) were compared by the Fisher's exact test for categorical variables; T test or nonparametric Mann–Whitney U test was adopted for continuous variables. Two-way ANOVA was used to assess the treatment effect over time in the overall population for hemodynamics and the biomarkers (hs-cTnT, NSE) which were considered in base 10 logarithm transformed. Sidak's post hoc correction for multiple comparisons was used for the comparison between groups at each time point. The sample size was estimated on the total number of defibrillations delivered prior to final resuscitation. From our data from a previous study^20^, assuming a reduction of defibrillations prior to resuscitation of at least 80% in the esmolol group compared to the control one, 8 animals per group would have been needed to have a power = 0.8 (a = 0.05, 2-sided). A value of *p* < 0.05 was considered significant. Data analyses were performed using GraphPad Prism (version 7.02 for Windows, GraphPad, Software, USA).

## Results

No significant differences in hemodynamics, EtCO2, CO, and arterial blood gases were observed between the 2 groups at baseline (Table 1). Seven animals in each group were successfully resuscitated. No significant differences were observed between groups in the duration of CPR, in the number of recurrent VFs within 15 min post-ROSC, and in the total number of defibrillations delivered prior to final resuscitation (Table 2).

**Table 1:** Hemodynamics and arterial blood gas analysis.

	Control n = 8	Esmolol n = 8	*p* value
**Heart Rate, beat/min**			
BL	98 ± 11	90 ± 8	0.56
1 h	190 ± 10	143 ± 11	0.02
2 h	171 ± 10	135 ± 12	0.05
3 h	167 ± 20	116 ± 13	0.05
4 h	153 ± 19	108 ± 13	0.08
**Systolic arterial pressure, mmHg**			
BL	116 ± 3	117 ± 4	0.82
1 h	102 ± 5	104 ± 8	0.84
2 h	101 ± 5	109 ± 6	0.37
3 h	96 ± 3	110 ± 7	0.11
4 h	93 ± 6	120 ± 4	0.004
**Mean arterial pressure, mmHg**			
BL	97 ± 4	99 ± 4	0.73
1 h	76 ± 4	86 ± 8	0.25
2 h	83 ± 6	95 ± 5	0.14
3 h	79 ± 6	93 ± 6	0.13
4 h	76 ± 4	103 ± 5	0.002
**Diastolic arterial pressure, mmHg**			
BL	84 ± 3	83 ± 5	0.89
1 h	62 ± 3	75 ± 7	0.13
2 h	72 ± 5	86 ± 5	0.08
3 h	68 ± 6	83 ± 6	0.12
4 h	63 ± 4	93 ± 5	0.001
**Right atrial pressure, mmHg**			
BL	6 ± 1	6 ± 1	0.75
1 h	9 ± 1	6 ± 1	0.11
2 h	8 ± 1	7 ± 1	0.31
3 h	7 ± 1	7 ± 1	0.47
4 h	8 ± 1	6 ± 1	0.21
**Mean pulmonary artery pressure,** **mmHg**			
BL	18 ± 2	19 ± 1	0.62
1 h	21 ± 2	20 ± 2	0.65
2 h	19 ± 2	20 ± 2	0.97
3 h	12 ± 1	21 ± 1	0.30
4 h	20 ± 1	21 ± 1	0.67
**Pulmonary Capillary Wedge Pressure, mmHg**			
BL	10 ± 1	8 ± 1	0.40
1 h	11 ± 2	9 ± 1	0.30
2 h	10 ± 1	8 ± 0	0.18
3 h	11 ± 1	11 ± 1	0.90
4 h	11 ± 1	10 ± 1	0.40
**LV Cardiac Output, L/min**			
BL	4 ± 0	4 ± 0	0.32
1 h	4 ± 0	4 ± 0	0.85
2 h	3 ± 0	3 ± 0	0.39
3 h	3 ± 0	3 ± 0	0.1
4 h	3 ± 0	3 ± 0	0.47
**Stroke volume, ml**			
BL	46 ± 4	45 ± 5	0.82
1 h	19 ± 2	25 ± 4	0.20
2 h	19 ± 2	22 ± 2	0.45
3 h	18 ± 4	26 ± 4	0.18
4 h	20 ± 3	33 ± 6	0.09
**pH**			
BL	7.48 ± 0	7.47 ± 0	0.79
2 h	7.34 ± 0	7.38 ± 0	0.41
4 h	7.40 ± 0	7.41 ± 0	0.70
**PaCO**_**2**_**, mmHg**			
BL	38 ± 1	37 ± 1	0.35
2 h	40 ± 1	40 ± 1	0.90
4 h	40 ± 0	41 ± 2	0.77
**PaO**_**2**_**, mmHg**			
BL	78 ± 6	79 ± 5	0.93
2 h	111 ± 8	109 ± 9	0.90
4 h	103 ± 13	111 ± 11	0. 63
**BE, mmol/L**			
BL	5 ± 2	3 ± 1	0.45
2 h	−3 ± 2	−2 ± 1	0.54
4 h	1 ± 2	2 ± 2	0.71
**HCO**_**3**_**, mmol/L**			
BL	29 ± 1	27 ± 1	0.36
2 h	22 ± 2	25 ± 2	0.36
4 h	25 ± 2	26 ± 1	0.68

Data are expressed as mean ± SEM. *p* values refer to Sidak's post hoc correction for multiple comparisons. BL, baseline; LV, left ventricle; PaO_2_, arterial partial pressure of oxygen; PaCO_2_, arterial partial pressure of carbon dioxide; BE, base excess.

**Table 2:** Resuscitation outcome.

	Control n = 8	Esmolol n = 8	*p* value
Defibrillations to ROSC, n	2 [1, 2]	1 [1–1.5]	0.43
Recurrent VFs within 15 min post ROSC, n	7 [0.5–19.5]	0 [0–9]	0.29
Duration of CPR prior to ROSC, sec	320 [300–62]	304 [301.5–307]	0.88
Total defibrillations delivered to final resuscitation, n	8 [2–29]	7 [1–12]	0.28
Successful resuscitation, n	7/8	7/8	> 0.99
96 h survival, n	4/7	4/7	> 0.99
96 h survival with good OPC score, n	2/7	3/7	> 0.99
**NAS**			
24 h	63 [38–88]	63 [21–100]	> 0.99
48 h	76 [50–100]	70 [30–100]	0.60
72 h	76 [53–96]	100 [40–100]	0.90
**NDS**			
24 h	116 ± 38	119 ± 53	0.97
48 h	65 ± 18	103 ± 50	0.56
72 h	60 ± 35	79 ± 56	0.79

Data are expressed as median and interquartile range or mean ± SEM.

ROSC, return to spontaneous circulation; CPR, cardiopulmonary resuscitation; OPC, Overall performance category (OPC was defined good when = 1–2); NAS, Neurological Alertness Score; NDS, Neuronal Deficit Score.

**Figure 2:**
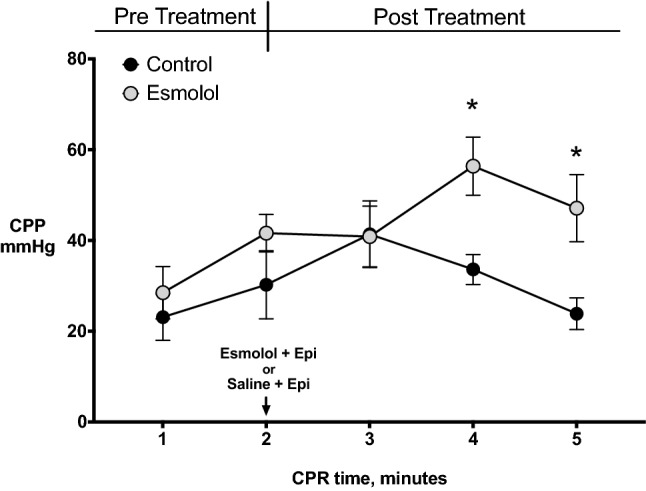
Coronary prefusion pressure (CPP) during cardiopulmonary resuscitation (CPR). Data are expressed as mean ± SEM. Repeated-measures two-way ANOVA was used to assess the treatment effect over time. *p < 0.05 vs. Control.

During the initial 2 min of CPR, CPP was equivalent in the 2 groups. However, following treatment administration, CPP was significantly higher in the esmolol group compared to the control one (*p* < 0.05, Fig. 2).

Post-resuscitation heart rate (HR) was lower in the esmolol group compared to the control group, with a significant reduction at 1 h after resuscitation (*p* = 0.017 Table 1). This was associated with significantly higher systolic, mean, and diastolic arterial pressures in animals treated with esmolol compared to those receiving placebo (Table 1). No differences in post-resuscitation pulmonary hemodynamics and cardiac function parameters were observed between the 2 groups, although a trend towards a higher stroke volume was observed in the esmolol group compared to the control one 4 h after resuscitation (*p* = 0.09, Table 1).

**Figure 3:**
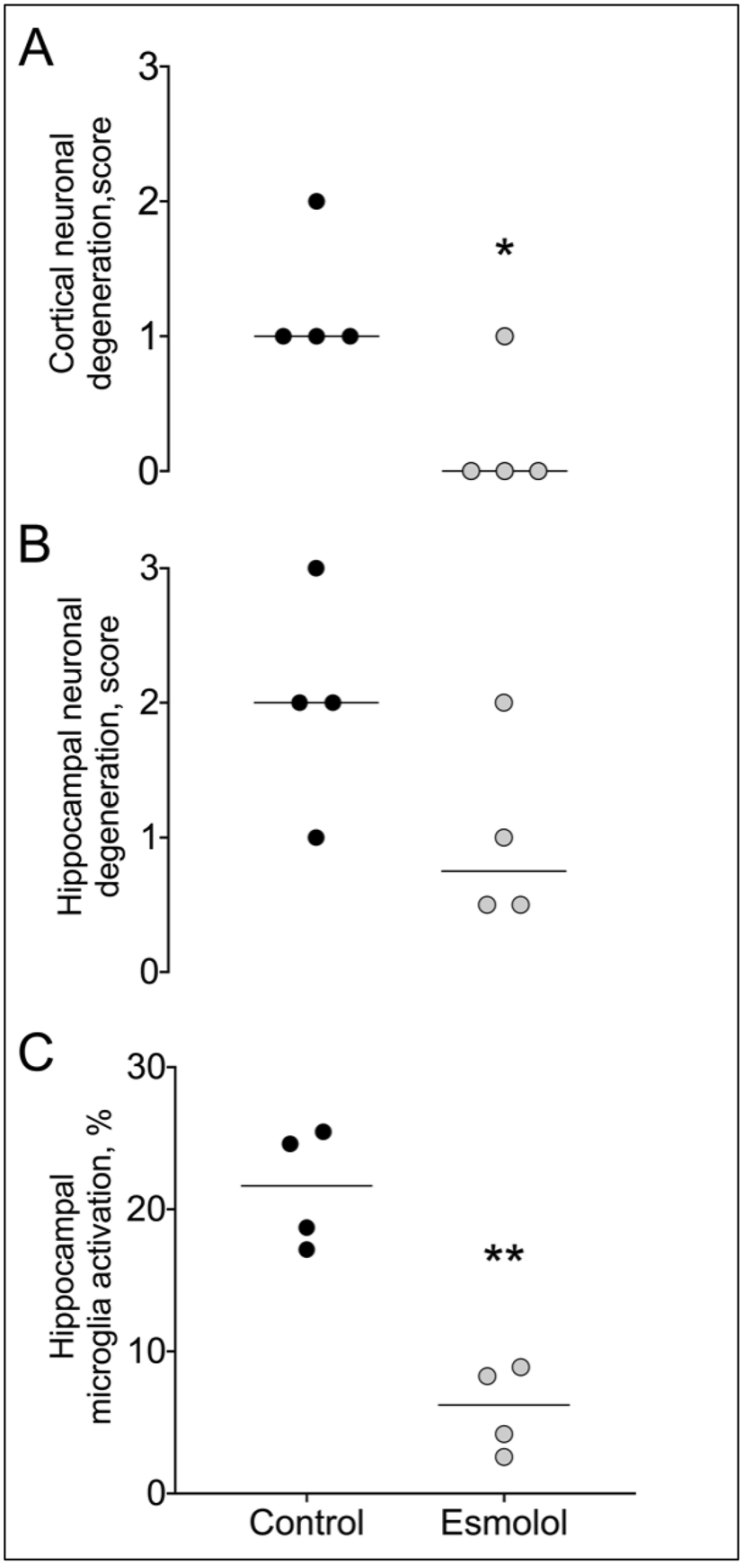
Brain histopathology. (**A**) Cortical and (**B**) Hippocampal neuronal degeneration/necrosis. (**C**) Microglia activation in the hippocampus. **p < 0.001 vs. Control.

**Figure 4:**
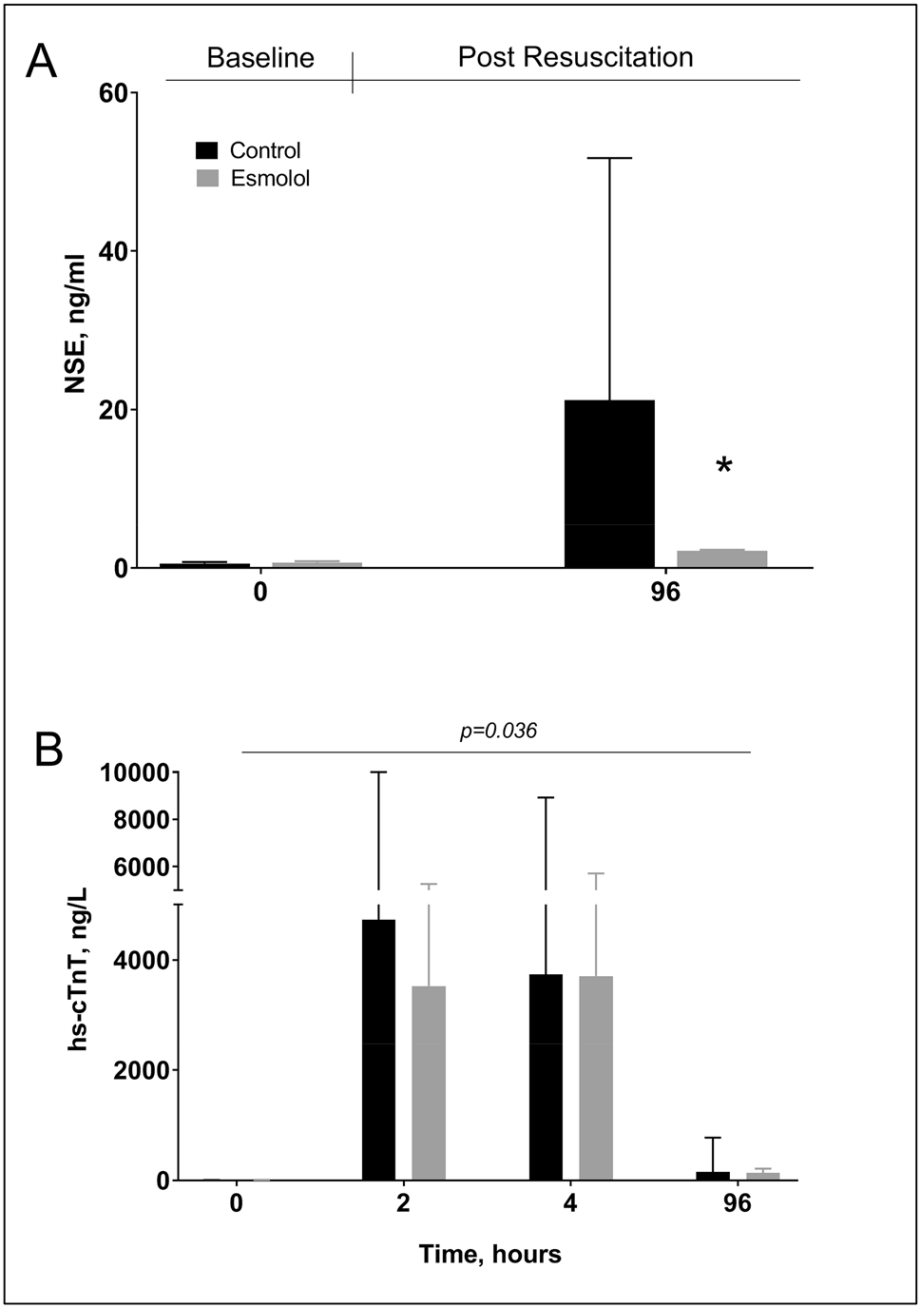
Serum neuronal specific enolase (NSE) levels at baseline (BL) and at 96 h after resuscitation (**A**). Plasma high-sensitive cardiac troponin T (hs-cTnT) levels at BL, 2, 4, and 96 h post-resuscitation (**B**). Data are reported as mean ± SEM. Two-way ANOVA: for NSE—treatment effect *p* = 0.0002, time effect *p* < 0.0001, interaction effect *p* = 0.0013; for hs-cTnT—treatment effect *p* = 0.036, time effect *p* < 0.0001, interaction effect *p* = 0.55. *p < 0.05 vs. Control.

There were no differences between groups in the number of animals that survived up to 96 h, and in good neurological recovery, although a trend in favor of esmolol was observed (43% vs. 29%, (Table 2). Brain histology showed a significant reduction in cortical neuronal degeneration/necrosis in the esmolol group compared to the control one (mean score 0.3 vs. 1.3, *p* = 0.03, Fig. 3A), while only a trend was present in the hippocampal region (mean score 1 vs. 2, *p* = 0.11, Fig. 3B). A marked reduction of microglial activation in the hippocampus was also observed after treatment with esmolol compared to control (mean % 6 vs. 2, *p* < 0.001, Fig. 3C). Significantly lower circulating levels of NSE were measured in esmolol animals compared to controls at 96 h after resuscitation (median 2.2 vs. 21, *p* < 0.0001, Fig. 4A).

Plasmatic levels of hs-cTnT were lower in the esmolol group compared to the control one up to 96 h after resuscitation (Fig. 4B, *p* = 0.036). However, macroscopic anatomy of the heart showed similar LV infarct size between the 2 groups (13% vs. 9%, *p* = 0.23).

## Discussion

This experimental study demonstrated that in a porcine model of ischemically induced CA, esmolol administration together with epinephrine during CPR reduced neurological injury, as represented by less neuronal degeneration, microglial activation, and lower circulating levels of NSE, compared to placebo with epinephrine. Esmolol in addition to epinephrine during CPR also accounted for a higher CPP compared to saline plus epinephrine and prevented post-resuscitation tachycardia and arterial hypotension. No effects on long term functional outcome were observed. However, the primary endpoint of the study was not demonstrated, as no difference in the number of defibrillations needed to resuscitation was detected between the groups.

The beneficial effects of esmolol in terminating VF have been reported in small retrospective case series including CA patients with refractory VF, indicating that ß1-blockade should be considered in this setting prior to cessation of resuscitative efforts^21,22^. This was not the case in our study, in which neuroprotection represented the main result after administration of esmolol during CPR. Neuroprotection with reduced hippocampal neuronal degeneration and smaller cortical infarct size by esmolol compared to control has been previously described after transient focal ischemia in rats^23,24^. We have also previously reported that epinephrine reduced cerebral cortical microvascular blood flow increasing the severity of cerebral ischemia during CPR in pigs, while beta-blockade, in association with an alpha-1 adrenergic antagonist, reverted this detrimental effect^25^. More recently, in a porcine model of prolonged VF, a bolus of esmolol during CPR has been proven to alleviate the impairment of cerebral microcirculation caused by the administration of epinephrine and to improve neurological outcome, in comparison to epinephrine alone^26^. Indeed, the density of perfused vessel was higher in esmolol-treated animals with a preserved morphology of brain endothelial cells, which appeared disrupted by epinephrine^26^. Thus, a better cortical microcirculation after esmolol may be hypothesized and likely accounted for the markedly reduced neuronal degeneration/necrosis together with neuroinflammation and NSE release observed in our study. Unfortunately, neuroprotection by combination of esmolol and epinephrine was observed only on histopathology and circulating biomarkers, while no effect on long term neurological recovery was found. One reason might be the relatively small sample of animals, attributable to many resuscitated pigs (42%) dying in the first days after resuscitation. These early deaths, while reinforcing the clinical relevance of this model of CA with an underlying AMI, obscured the neurological outcome in the dead animals. Although the same mortality rate was observed in both groups, if animals died due to the AMI, as it may be extrapolated by the greater hs-cTnT release in the control group, or due to a more severe brain damage, cannot be clarified.

Another possible explanation for the neuroprotective effect of β1-blockade observed during CPR might be the higher CPP recorded after esmolol administration compared to placebo, which might have accounted for a higher cerebral perfusion and consequent prevention of neuronal injury. Previous experimental CPR studies showed the same increase in CPP starting 1 min after esmolol administration and maintained throughout resuscitation compared to placebo^13,27^. Esmolol would likely decrease the oxygen requirements of the myocardium during VF, further exacerbated by epinephrine, and thereby minimize global ischemic injury and ATP content reduction. This might mitigate the evolution towards the condition of stone heart, consequently improving left ventricle compliance and stroke volume generated by chest compression and ultimately CPP, compared to epinephrine alone^8,28^. We also observed that post-resuscitation tachycardia and arterial hypotension were prevented by esmolol, likely contributing to a better post-ROSC cerebral perfusion, further preserving the brain.

Multiple studies in models of electrically induced VF demonstrated that esmolol given during CPR seemed to modulate the electrical properties of the myocardium and intracellular calcium handling, which contributed to the reduction in the occurrence of recurrent and resistant VFs and to a better post-resuscitation myocardial function^8,11,14,15^. Whether the cardioprotective effect of esmolol is still maintained in the instance of ischemically-induced CA remains yet to be elucidated. Indeed, no improvement in cardiac output or reduction of myocardial infarct size has been observed after a single dose of esmolol prior to adrenaline compared to placebo in a recent study employing an ischemic CA pig model with extracorporeal resuscitation^17^. In our model, myocardial protection was suggested by the reduced levels of circulating hs-cTnT observed after beta-blocked administration compared to the use of placebo. However, LV infarct size was similar in the two groups and VF was not terminated by a lower number of defibrillations in esmolol-treated animals compared to controls, as initially hypothesized. Whether starting a continuous infusion of esmolol after the initial bolus or first ROSC would have been more effective in preventing recurrency of VF and achieving sustained ROSC than the single bolus remains unknown. Nevertheless, the single bolus injection strategy was chosen for its high translational and practical application in the clinical out-of-hospital settings.

Other limitations should be acknowledged. Firstly, this was a purely descriptive study. Indeed, earlier experimental studies have already compared epinephrine with other different vasopressor treatments during CPR, i.e. noradrenaline, vasopressin, epinephrine associated with selective/non-selective beta blockers, amines with predominant alpha-1 adrenergic actions, alpha-2 adrenergic agonists, consistently reporting the greater myocardial oxygen consumption after epinephrine as the main mechanisms accounting for post-resuscitation myocardial dysfunction and anticipating more optimal vasopressor interventions^8,13,16,29–33^. Unfortunately, among the above interventions, those investigated clinically, i.e. norepinephrine and vasopressin, failed to improve outcome compared to epinephrine^34,35^. More specifically, esmolol administered with epinephrine during CPR has been consistently shown to reduce myocardial dysfunction and to increase the rate of ROSC in contrast to epinephrine alone in the electrically induced VF, and mechanisms have been already described^13–16^. Thus, the present study was designed to confirm these beneficial effects of esmolol when added to epinephrine in a model of ischemically induced CA, while concurrent comparison with other vasopressors was not replicated. Secondly, since the original endpoint of this study was VF termination and not neurological outcome, no measures of cerebral perfusion pressure or carotid blood flow or regional brain oxygenation were planned to directly investigate and/or provide specific mechanistic insights of neuroprotection. Nevertheless, earlier studies have already described improved cerebral perfusion when beta-blockade was associated with epinephrine during CPR^25,26^. Thirdly, since the sample size was calculated considering the above primary endpoint, although the experimental model reproduced a clinically relevant condition with ischemic VF and a prolonged no-flow time, the number of animals in each group was not powered to detect a significant difference in long-term neurological outcomes. Fourthly, we are aware that the optimal hemodynamics usually observed in models of CPR performed in the laboratory, i.e. high CPP values after a prolonged untreated CA, can be hardly reproduced during a clinical resuscitation. Finally, post-resuscitation myocardial function was not directly assessed, i.e. through echocardiography; however, data on HR, hemodynamics, stroke volume, and circulating biomarkers confirm evidence of cardioprotection.

## Conclusions

In this preclinical model of ischemically induced CA, β1-blockade during CPR did not facilitate VF termination but provided neuroprotection. Indeed, esmolol administration together with epinephrine during CPR accounted for a reduction of neurological injury as represented by less neuronal degeneration and microglial activation, and lower levels of NSE compared to the control treatment with saline plus epinephrine.
